# Lycopene's Role in Mitigating Obesity‐Induced Cardiac Remodeling: Insights Into Inflammatory and MMP‐2 Pathways

**DOI:** 10.1002/mnfr.70395

**Published:** 2026-01-26

**Authors:** Carol Cristina Vagula de Almeida de Silva, Artur Junio Togneri Ferron, Fabiane Valentini Francisqueti‐Ferron, Alexandre Ribeiro da Silva, Silmeia Garcia Zanati Bazan, Jéssica Leite Garcia, Dijon Henrique Salomé de Campos, Mariane Róvero Costa, Daniele Dantas, Heloysa Amaral da Silva, Janaina Paixão das Chagas Silva, Giselle Pinto Faria de Lopes, Bertha Furlan Polegato, Camila Renata Correa, Fernando Moreto, Ana Lucia Anjos Ferreira

**Affiliations:** ^1^ Medical School Sao Paulo State University (UNESP) Botucatu Brazil; ^2^ Instituto de Estudos do Mar Almirante Paulo Moreira Marine Biotechnology Department IEAPM Rio de Janeiro Brazil; ^3^ Harvard Medical School Boston United States; ^4^ Institute of Biosciences Sao Paulo State University (UNESP) Botucatu Brazil; ^5^ Federal University of Paraná (UFPR) Curitiba Brazil

**Keywords:** cardiac remodeling, collagen I, inflammation, lycopene, metalloproteinase‐2, NF‐κB, obesity, TIMP‐2, TLR‐4

## Abstract

Obesity, characterized by chronic low‐grade inflammation, promotes cardiac structural and functional abnormalities. This study evaluated the therapeutic potential of lycopene in attenuating obesity‐induced cardiac remodeling, based on its antioxidant and anti‐inflammatory properties and its ability to inhibit matrix metalloproteinase‐2 (MMP‐2) activation, thereby preserving myocardial collagen integrity. Male Wistar rats were fed a high‐sugar, high‐fat (HSF) diet to induce obesity and cardiac remodeling. After the onset of cardiac dysfunction, animals received lycopene supplementation (10 mg/kg/day) for 10 weeks. The HSF diet caused metabolic disturbances, including hypertension, increased adiposity, and insulin resistance, accompanied by myocardial remodeling, inflammation, and elevated MMP‐2 activity. Lycopene supplementation reversed insulin resistance, mitigated myocardial remodeling, and improved both systolic and diastolic cardiac function. It also reduced inflammatory markers (TNF‐α, IL‐6, NF‐κB, TLR‐4), decreased MMP‐2 activation, and enhanced TIMP‐2 and type I collagen expression. Lycopene demonstrated cardioprotective and anti‐inflammatory effects in obesity‐induced cardiac remodeling. By targeting inflammation and extracellular matrix degradation, lycopene may serve as an effective adjunctive therapeutic approach for preventing or treating obesity‐related cardiac disorders.

## Graphical abstract

**A**. Consumption of a highsugar‐fat diet causes excess nutrients, hypertrophy and hyperplasia of adipocytes, causing obesity. Obesity leads to increased inflammatory levels in the heart, which stimulates greater activation of Metalloproteinase‐2 (MMP‐2), which has collagenolytic activity, degrading the collagen network in the heart, ultimately causing pathological cardiac remodeling, with systolic and diastolic dysfunction. **B**. Lycopene supplementation in obese individuals rat attenuates inflammation, MMP‐2 activity, and collagen degradation in the heart, and consequently cardiac remodeling, improving systolic and diastolic function.

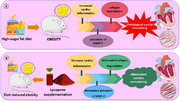

**Abbreviations**
A’: Late diastolic of the mitral annulus
AD: Aorta diameter
BF: Body fat
BW: Body weight
CI: Caloric intake
COL: Collagen
COL‐I: Collagen type I
Dec. time: Deceleration time
DTIS: Diastolic thickness of the intraventricular septum
E: Transmitral flow early peak velocity
E’: Early diastolic of the mitral annulus
E/E’: Ratio by the waves E and E’
EF: Ejection fraction
ECM: Extracellular matrix
FC: Feed consumption
FE: Feed efficiency
HFpEF: Heart failure with preserved ejection fraction
HOMA‐IR: Homeostatic model of insulin resistance
HPLC: High performance liquid chromatography
HR: Heart rate
HSF: High sugar fat
IL‐6: Interleucin‐6
IP: Intraperitoneal
IRT: Isovolumetric relaxation time
LA: Left atrium
LV: Left ventricle
LVDD: Left ventricle diastolic diameter
LVPWT: Left ventricle diastolic thickness posterior wall
LVSD: Left ventricle systolic diameter
LY: Lycopene
MAPK: Mitogen‐activated protein kinase
MCP‐1: Monocyte chemoattracting protein ‐ 1
MMP: Metalloproteinase
MMP‐2: Metalloproteinase‐2
NF‐κB: Nuclear Factor Kappa B
pNF‐κB: Phosphorylated nuclear factor kappa B
PWSV: Posterior wall shortening velocity
RAAS: Renin‐angiotensin‐aldosterone system
RDI: Recommended daily intake
RTLV: Relative thickness of the left ventricle
SBP: Systolic blood pressure
TIMP: Tissue inhibitor of metalloproteinases
TIMP‐2: Tissue inhibitor of metalloproteinases ‐ 2
TLR‐4: Toll‐like receptor‐4
TNF‐α: tumor Necrosis factor‐α

## 1 Introduction

Obesity, a growing global health crisis, is closely associated with a spectrum of cardiovascular complications, particularly profound structural and functional changes in the heart, collectively referred to as cardiac remodeling [1, 2]. This pathological progression, characterized by alterations in myocardial architecture and function, is a strong predictor of adverse cardiovascular events and increased mortality [3]. Experimental hypercaloric diet models consistently induce myocardial dysfunction in obese rodents, closely mirroring clinical observations [4, 5]. Although adipose tissue accumulation is associated with cardiac impairments, the molecular mechanisms underlying these changes remain not yet fully understood and are the focus of ongoing investigation. Growing evidence implicates chronic low‐grade inflammation as a central pathogenic factor in the development of obesity‐induced cardiac remodeling [6, 7].

The complex interplay between obesity and inflammation is often initiated by Toll‐like receptor 4 (TLR‐4) activation. This pattern recognition receptor is activated by endogenous ligands, such as saturated fatty acids [6]. Upon activation, TLR‐4 triggers downstream signaling cascades, leading to the activation of nuclear factor‐kappa B (NF‐κB), a key transcriptional regulator of inflammatory gene expression [8]. This activation, in turn, triggers the transcriptional upregulation and subsequent release of a diverse array of pro‐inflammatory cytokines such as tumor necrosis factor‐alpha (TNF‐α), interleukin‐6 (IL‐6), and monocyte chemoattractant protein‐1 (MCP‐1) [8]. These proinflammatory mediators subsequently disrupt extracellular matrix homeostasis within the myocardium. This imbalance results mainly from increased activation of matrix metalloproteinases (MMPs), particularly MMP‐2 [9]. MMP‐2 is a key enzyme responsible for collagen degradation and plays a central role in the pathological remodeling of the cardiac extracellular matrix. Its excessive activation leads to interstitial collagen breakdown, ultimately contributing to adverse cardiac remodeling [9]. Given the prominent and complex role of inflammation in the pathogenesis of obesity‐related cardiovascular complications, therapeutic strategies specifically targeting inflammatory pathways have attracted significant scientific and clinical interest [4, 6, 10, 11].

Lycopene, a major dietary carotenoid, is a lipophilic hydrocarbon pigment abundant in red, pink, and orange fruits and vegetables (including tomatoes, red bell peppers, apricots, melons, papayas, grapes, peaches, watermelons, cranberries, and their derived products) and has emerged as a compelling natural compound with potent antioxidant and anti‐inflammatory properties. [12]. Although not formally classified as an essential nutrient, a growing body of evidence suggests that adequate dietary intake of lycopene is important to achieve its potential health benefits [13, 14, 15]. Numerous in vitro and in vivo investigations have consistently demonstrated lycopene's capacity to exert significant anti‐inflammatory actions, primarily through its ability to modulate transcriptional factors such as NF‐κB [16, 17, 18]. The cardioprotective attributes of lycopene have been extensively explored across various contexts of cardiac dysfunction [19, 20, 21, 22, 23]. However, there is a notable gap in the literature regarding the specific role of lycopene in modulating MMP‐2 activity in obesity‐induced cardiac remodeling. Consequently, this study was designed to ascertain whether lycopene supplementation could effectively regulate cardiac remodeling in the context of obesity by directly targeting and modulating MMP‐2 activity. We hypothesized that lycopene supplementation in animals with obesity‐related cardiac remodeling would modulate the inflammatory response and attenuate disease progression by preserving extracellular matrix integrity.

## 2 Experimental Section

### 2.1 Animals and Experimental Protocol

Male Wistar rats (*Rattus norvegicus* (Berkernhout, 1769)) (∼200 g, *n* = 32) were initially randomized into two experimental groups, in which one received the control diet (C, *n* = 16) and the other group received high sugar‐fat diet (HSF, *n* = 16) for 20 weeks. Diets and water were provided ad libitum. The diet composition used here has been previously described [24, 25]. All the animals were kept in a controlled environment (22°C ± 3°C, 12 h light‐dark cycle and relative humidity of 60 ± 5%). The sample size was determined based on previous studies [24, 25, 26]. All the experiments were performed in accordance with the National Institute of Health Guide for the Care and Use of Laboratory Animals, and the procedures were approved by the Animal Ethics Committee of Botucatu Medical School (#1272/2018).

At the 20th week of HSF diet intake (Group HSF), the cardiac dysfunction was detected by echocardiography. Then, the animals were divided to begin the treatment with lycopene or corn oil, totaling four groups: Control (C, *n* = 8); Control supplemented with lycopene (tomato oleoresin) (C + Ly, *n* = 8); HSF (*n* = 8) and HSF supplemented with lycopene (HSF + Ly, *n* = 8). Lycopene was mixed with corn oil corresponding to 10 mg lycopene/kg of body weight (BW)/day and was given orally daily, in the morning, for a 10‐week period [27, 28]. To avoid differences in energy intake, all groups received the same amount of corn oil (approximately 2 mL/kg BW/day). The supplementation period and dose were chosen based on previous studies [19, 27, 28, 29]. At the end of the experiment (30th week), the animals were euthanized under deep anesthesia (thiopental 120 mg/kg BW, intraperitoneal (IP)) and decapitated to obtain blood and heart (LV, left ventricle) samples. Blood and LV were collected and appropriately stored for the following analyses: a) blood (lycopene, glucose, insulin concentrations); b) heart (lycopene and inflammatory cytokines concentration; metalloproteinase‐2 activity; protein expression of TLR‐4, NF‐κB, pNF‐κB, MMP‐2, TIMP‐2, and collagen type I).

### 2.2 Diets

The diets used in this study were prepared in the laboratory following an obesity induction model [4]. The HSF diet contained soybean meal, sorghum, soybean peel, dextrin, sucrose, fructose, lard, vitamins, and minerals, with an additional 25% sucrose in the drinking water. Control diet contained soybean meal, sorghum, soybean peel, dextrin, soybean oil, vitamins, and minerals. The nutrients and nutritional composition of each diet are presented in Table 1.

**TABLE 1:** Diet composition and nutritional values.

Components (g/kg)	Control	HSF
Soybean meal	335.0	340.0
Sorghum	278.0	80.0
Soy hull	188.0	116.0
Dextrin	146.0	20.0
Sucrose	0.0	80.0
Fructose	0.0	180.0
Soybean oil	14.0	0.0
Lard	0.0	154.0
Minerals	25.0	25.0
Salt	4.0	8.0
**Nutritional values**		
Protein (% of ingredients)	20.0	18.0
Carbohydrate (% of ingredients)	60.0	53.5
Fat (%of ingredients)	4.0	16.5
% of unsaturated fat	69.0	47.0
% of saturated fat	31.0	53.0
% Energy from protein	22.9	16.6
% Energy from fat	10.4	34.2
Energy (kcal/g)	3.6	4.4

Abbreviation: HSF, high sugar‐fat diet.

### 2.3 Lycopene Preparation

Lycopene (Lyc‐O‐Mato 6% dewaxed; LycoRed Natural Products Industries, Beersheba, Israel) was mixed with corn oil and, due to its photosensitivity [30], kept in the dark at 4°C to prevent photodegradation until use [25, 31]. The lycopene‐corn oil mixture was kept for 20 min in a water‐bath at 54°C before administration to the animals. The total amount of lycopene in each solution was 5 mg/mL. Lycopene stability was confirmed by diode‐array spectra at 450 nm, as previously described [32].

### 2.4 Dosage Information

Lycopene supplementation was administered daily, in the morning, via oral gavage, at a dose of 10 mg lycopene/kg BW/day, for a duration of 10 weeks. This specific dosage and treatment period were selected based on their demonstrated efficacy in previous studies utilizing models of cardiovascular and metabolic diseases [19, 27, 28, 29]. To determine the human equivalent dose (HED), a conversion factor of 0.162 [33] for Wistar rats derived from body surface area normalization, was applied. Consequently, the 10 mg/kg BW/day dose in rats approximates 1.62 mg/kg BW/day in humans. For an average 70 kg adult, this translates to an approximate daily intake of 113.4 mg of lycopene. Although this dose exceeds typical dietary lycopene intakes (often estimated at 5–10 mg/day for general health benefits, noting that no official recommended daily intake (RDI) has been established), it remains achievable through commercially available supplements, which frequently contain elevated lycopene concentrations, or via a diet rich in concentrated tomato products.

### 2.5 Nutritional Evaluation

Nutritional evaluation included: feed consumption (FC) as daily consumed amount in grams of chow feed; final body weight (BW); caloric intake (CI) calculated according to the following formula for the control group: caloric intake (kcal/day) = feed consumption (g) ×dietary energy (3.59 kcal/g) [4, 25, 31, 34]. For the HSF group, the caloric intake was calculated as following: water volume consumed (mL) × 0.25 (equivalent to 25% fructose) × 4 (calories per gram of carbohydrate) + caloric intake providing by the chow (feed consumption (g)) *x* dietary energy (4.35 kcal/g) [4, 25, 31, 34]. The adiposity index, considered an obesity marker, was calculated as follows: adiposity index = (total body fat (BF)/final body weight) × 100 [4, 25, 31, 34]. BF was evaluated considering the sum of the individual fat weights: BF = epididymal fat + retroperitoneal fat + visceral fat [4, 25, 31, 34].

### 2.6 Metabolic and Hormonal Analyses

After an 8‐h fast, blood samples were collected for biochemical analysis. Blood glucose levels were measured using a glucometer (Accu‐Chek Performa; Roche Diagnostics, Indianapolis, IN, USA). The insulin levels were analyzed on plasma by ELISA assay with commercial kits (Millipore) [25]. The HOMA‐IR (homeostatic model of insulin resistance), considered an insulin resistance index, was calculated by the following formula: HOMA‐IR = [fasting glucose (mmol/L) × fasting insulin (µU/mL)]/22.5 [4].

### 2.7 Systolic Blood Pressure (SBP) Measurements

SBP was evaluated by a noninvasive tail‐cuff method with a NarcoBioSystems Electro‐Sphygmomanometer (International Biomedical, Austin, TX, USA) in conscious rats. To perform this, the animals were heated during 4–5 min in a wooden box (50 × 40 cm), with two incandescent lamps and temperature between 38 and 40°C, to induce arterial vasodilation in the tail. Then, the rats were transferred to an iron cylindrical support specially made to allow the total exposure of the animal's tail [19]. After this procedure, a cuff with a pneumatic pulse sensor was attached to the tail and inflated to 200 mmHg pressure and successively deflated. Blood pressure values were documented on a Gould RS 3200 polygraph (Gould Instrumental Valley View, Cleveland, OH, USA). The final SBP of each animal considered the average of three pressure readings.

### 2.8 Lycopene Uptake and Absorption

The presence of lycopene was determined in plasma and cardiac tissue homogenates as previously reported [35]. The presence of lycopene was determined in plasma and cardiac tissue homogenate. To extract the carotenoids, samples were incubated with internal standard (equinenone), chloroform/methanol CHCl_3_/CH_3_OH (3 mL, 2:1, v/v) and 500 mL of saline 8.5 g/L. Then the samples were centrifuged at 2000× g for 10 min and the supernatant was collected and hexane was added. The chloroform and hexane layers were evaporated under nitrogen, and the residue was resuspended in 150 mL of ethanol and sonicated for 30 s. 50 µL of this aliquot was injected into the HPLC. The HPLC system was a Waters Alliance 2695 (Waters, Wilmington, MA, USA) and consisted of pump and chromatography bound to a 2996 programmable photodiode array detector, a C30 carotenoid column (3 mm, 150 × 3 × 4.6 mm, YMC,Wilmington, MA, USA) and Empower 3 chromatography data software (Milford, MA, USA). The HPLC system programmable photodiode array detector was set at 450 nm for carotenoids. The mobile phase consisted of ethanol/methanol/methyl‐*tert*‐butyl ether/water (83:15:2, v/v/v, 15 g/L with ammonium acetate in water, solvent A) and methanol/methyl‐tert‐butyl ether/water (8:90:2, v/v/v, 10 g/L with ammonium acetate in water, solvent B). The gradient procedure, at a flow rate of 1 mL/min (16°C), was as follows: (1) 100% solvent A was used for 2 min followed by a 6 min linear gradient to 70% solvent A; (2) a 3 min hold followed by a 10 min linear gradient to 45% solvent A; (3) a 2 min hold, then a 10 min linear gradient to 5% solvent A; (4) a 4 min hold, then a 2 min linear gradient back to 100% solvent A. For the quantification of the chromatograms, a comparison was made between the area ratio of the substance and area of the internal standard obtained in the analysis [29].

### 2.9 Echocardiographic Study

One day before euthanasia, all animals were evaluated in vivo by transthoracic echocardiography, with a Vivid S6 system equipped with multifrequency ultrasonic transducer 5.0 to 11.5 MHz (General Electric Medical Systems, Tirat Carmel, Israel). The animals were lightly anesthetized by intraperitoneal injection with a mixture of ketamine (50 mg/kg BW) and xylazine (1 mg/kg BW), put in left decubitus position and only one examiner made all the exams. All the analyses made by the examiner were blinded. The heart image structural measurements were obtained in one‐dimensional mode (M‐mode) guided by the images in two‐dimensional mode with the transducer in the parasternal position, minor axis. Left ventricular (LV) evaluation was performed with the cursor M‐mode just below the mitral valve plane at the level of the papillary muscles. The aorta and left atrium images were obtained by positioning the M‐mode course to plan the aortic valve level [25]. The following cardiac structures were evaluated: diastolic diameter (LVDD); systolic (LVSD) LV; left ventricle diastolic thickness posterior wall (LVPWD); aorta diameter (AD); left atrium (LA). The LV diastolic function was assessed by the transmitral flow at early peak velocity (E). The LV systolic function was evaluated by ejection fraction and posterior wall shortening velocity (PWSV). The joint assessment of diastolic and systolic LV function was performed using the Tei index (sum of isovolumetric contraction and IRT time, divided by the left ventricular ejection time). The study was complemented by tissue Doppler evaluation, considering early diastolic (E’), and late (A’) of the mitral annulus (arithmetic average travel speeds of lateral and septal walls), and the ratio by the waves E and E’ (E/E’).

### 2.10 Inflammatory Cytokines Analyses

The tissue samples were homogenized in phosphate‐buffered saline (PBS) at a ratio of 1:10 (sample:PBS). Cardiac levels of MCP‐1, TNF‐α, and IL‐6 were measured by ELISA purchased from R&D Systems. A microplate spectrophotometer reader (SpectraMax 190; Molecular Devices) was used according to the manufacturer's instructions.

### 2.11 Metalloproteinase‐2 Activity Determination

Cardiac tissue samples were diluted and homogenized in lysis buffer (50 mM Tris, pH 7.4, 0.2 M NaCl, 0.1% Triton‐X, and 10 mM CaCl_2_), then subjected to zymography according to previously described conditions [36]. The gel was photographed and the intensity of the gelatinolytic action (light bands) was analyzed on a White Darkhon UV‐UVP image analyzer. Quantitative analyses of the protein bands (blots) were performed by the Gel Logic 6000 Pro photodocumenter (Carestream Health, Rochester, NY, USA). Results were expressed as MMP‐2 activity.

### 2.12 Protein Expression of Toll‐Like Receptor‐4 (TLR‐4), Nuclear Factor Kappa B (NF‐κB), Phosphorylated Nuclear Factor Kappa B (pNF‐κB), Metalloproteinase‐2 (MMP‐2), Tissue Inhibitor of Metalloproteinases 2 (TIMP‐2), and Collagen Type I (Col I) by Western Blot Analysis

The protein concentration of the LV extract (including the cell membrane, cytoplasm, and nucleus) was determined by the Bradford method [37]. Samples of LV (50 µg protein) were homogenized in RIPA buffer, diluted in Laemmli buffer, and heated at 100°C for 5 min before being loaded onto a 10% SDS–polyacrylamide gel. The samples were transferred to a nitrocellulose membrane in methanol. Incubation with the primary antibodies (Santa Cruz Biotechnology and Abcam) was performed overnight at 4°C in Tris‐buffered saline solution containing Tween 20 (TBS‐T) and 3 % nonfat dried milk. Antibody dilutions were: 1:200 for mouse anti‐TLR‐4 sc293072, 1:200 for rabbit anti‐β‐actin ab8227, 1:100 for rabbit anti‐ pNF‐κB (ser536) sc33020, 1:200 for mouse antitotal NF‐κB sc8008, 1:100 for mouse anti‐MMP‐2 sc13595, 1:100 for mouse anti‐TIMP‐2 sc21735, 1:100 for mouse anti‐Type I collagen sc59772. After overnight incubation at 4°C in TBS‐T containing 1 % nonfat dried milk with the Abcam secondary antibodies (dilution 1:10 000) anti‐rabbit ab97069 and anti‐mouse ab98808, protein was revealed using the chemiluminescence method according to the manufacturer's instructions (ECL SuperSignal West Pico Chemiluminescent Substrate; Thermo Scientific). Band intensities were evaluated using Gel‐Pro Analyzer (Media Cybernetics).

### 2.13 Histological Characterization

The LV dissected from 8 animals of each experimental group were fixed in 10% phormaldehyde solution for 24 h. Then staining was conducted as previously reported [X]. Briefly, after deparaffinization and rehydration of the tissues, the Harris hematoxylin and eosin staining was performed with three serial slides of each animal. To ensure reproducibility, all cases were examined by two observers, and all morphological observations were made using at least ten randomly selected fields at 20, 40, and 63‐fold magnification. Images were captured using an optical microscope, attached to a video camera, connected to a computer.

### 2.14 Statistical Analysis

The results were expressed in mean ± standard deviation (SD). Two‐way analysis of variance (ANOVA) was used to determine the differences among the groups (Diet and Treatment) and the interaction between them (Diet x Treatment). Then, the Tukey's posthoc test for multiple comparisons was performed when significant interactions between the grouping variables (Diet x Treatment) was found (*p* < 0.05). All the statistical analyses were performed using SigmaStat for Windows (Version 3.5, San Jose, CA, USA).

## 3 Results

From the tomato oleoresin corn oil mixture, the animals received 10 mg/kg body weight per day of total lycopene. There was no detectable lycopene in the plasma or heart of the control or HSF groups due to the absence of lycopene in their diets [35]. After 10 weeks of tomato oleoresin supplementation, the plasma levels of lycopene reached 3.6 ± 0.5 nnng/dL in the C + Ly and 6.8 ± 2.3 mg/dL in the HSF + Ly groups, while cardiac levels were 4.8 ± 2.3 ng/g of tissue of tissue in C+Ly and 2.4 ± 0.3 ng/g of tissue in HSF+Ly groups.

The HSF group displayed higher caloric intake (kcal/day), final body weight (g), adiposity index, glucose levels, insulin, HOMA‐IR, and systolic blood pressure values, as compared to the control group. Lycopene supplementation was associated with decreased insulin and HOMA‐IR, considering HSF groups only (HSF+Ly vs. HSF). No effect of lycopene was observed on the other nutritional and metabolic parameters (Table 2).

**TABLE 2:** Nutritional and metabolic parametersin obese rats.

	Groups				Effect		
Variables	C	C + Ly	HSF	HSF + Ly	Diet	Ly	Interaction
Body weight (g)	507 ± 21	514 ± 22	535 ± 21	536 ± 22	0.259	0.838	0.887
Adiposity index (%)	3.9 ± 0.4	4.3 ± 0.4	8 ± 0.4*	8.7 ± 0.4	0.001	0.222	0.715
SBP (mmHg)	121 ± 3	119 ± 4	137 ± 3*	146 ± 4	0.001	0.398	0.223
Caloric intake (Kcal/dia)	69 ± 9	131 ± 10*	121 ± 9*	119 ± 9#	0.048	0.004	0.002
Glucose (mg/dL)	77 ± 4	90 ± 5	97 ± 4*	105 ± 6	0.002	0.051	0.613
Insulin mg/dL	0.9 ± 0.2	1.3 ± 0.2*	5.6 ± 0.2*	1.4 ± 0.2#	0.001	0.001	0.001
HOMA‐IR	0.15 ± 0.07	0.30 ± 0.08*	1.37 ± 0.07*	0.29 ± 0.08#	0.001	0.001	0.001

*Note*: Data are expressed in mean ± standard deviation (*n* = 8 animals/group). Comparison by Two‐way ANOVA with Tukey post‐hoc (*p* < 0.05 Results followed by * indicate a significant difference when compared to the control group, and results followed by # indicate a significant difference when compared to the HSF group).

The high sugar fat treatment was associated with a significant cardiac remodeling (increased LVSD, LVPWD, and reduced LVDD), and deterioration of both systolic (decreased ejection fraction, Tei‐a and Tei‐b) and diastolic (increased E/E’ and decreased Tei‐a and Tei‐b) functions as compared to control group. Lycopene supplementation was associated with significant improvement in several remodeling, systolic, and diastolic functional parameters (LVSD, DTIS, EF, RTLV, and E/E’) (HSF vs. HSF+Ly). There was a significant interaction between diet (HSF) and lycopene treatment for LVSD, DTIS, EF parameters (Table 3).

**TABLE 3:** Morphological and functional study evaluated by echocardiography in obese rats.

	Groups				Effect		
Variables	C	C + Ly	HSF	HSF + Ly	Diet	Ly	Interaction
LVDD (mm)	7.0 ± 0.1	7.0 ± 0.1	6.7 ± 0.1*	6.9 ± 0.1	0.035	0.410	0.402
LVSD (mm)	2.75 ± 0.08	2.81 ± 0.1*	3.21 ± 0.08*	2.77 ± 0.1^#^	0.032	0.048	0.012
LVPWD (mm)	1.59 ± 0.03	1.56 ± 0.04	1.71 ± 0.04	1.61 ± 0.04	0.061	0.118	0.404
DTIS (mm)	1.72 ± 0.03	1.61 ± 0.04*	1.93 ± ± 0.03*	1.67 ± 0.04^#^	0.012	0.001	0.028
AD (mm)	3.87 ± 0.05	3.9 ± 0.06	3.9 ± 0.06	3.86 ± 0.06	0.929	0.937	0.622
LA (mm)	4.97 ± 0.09	4.94 ± 0.11	5.03 ± 0.09	4.8 ± 0.11	0.587	0.758	0.092
RTLV	0.45 ± 0.01	0.44 ± 0.01	0.51 ± 0.01*	0.46 ± 0.01	0.005	0.057	0.217
HR (bpm)	256 ± 13	266 ± 16	263 ± 14	262 ± 16	0.930	0.758	0.715
E (cm/s)	73 ± 1	72 ± 2	77 ± 2	73 ± 2	0.392	0.299	0.519
PWSV (cm/s)	59 ± 1	61 ± 1	56 ± 1	59 ± 1	0.191	0.082	0.639
Dec. Time (ms)	46 ± 1	41 ± 1*	49 ± 1	43 ± 1	0.145	0.004	0.745
Tei‐a (ms)	115 ± 2	117 ± 2*	100 ± 2*	111 ± 2	0.001	0.039	0.123
Tei‐b (ms)	87 ± 2	93 ± 3*	78 ± 2*	85 ± 3	0.011	0.046	0.987
EF(%)	0.94 ± 0.007	0.93 ± 0.009*	0.88 ± 0.07*	0.93 ± 0.009^#^	0.002	0.017	0.003
E/E’	13.2 ± 0.4	12.7 ± 0.5*	15.3 ± 0.4*	13.7 ± 0.5^#^	0.003	0.042	0.254

*Note*: Data are expressed in mean ± standard deviation (*n* = 8 animals/group). Comparison by Two‐way ANOVA with Tukey post‐hoc (*p* < 0.05):Results followed by * indicate a significant difference when compared to the control group, and results followed by # indicate a significant difference when compared to the HSF group.

Abreviations: AD, aorta diameter; Dec. time, deceleration time; DTIS, diastolic thickness of the intraventricular septum; E, E‐wave peak transmitral early diastolic inflow velocity; EF, ejection fraction; E/E’, Ratio by the waves E and E'; HR, heart rate; LA, left atrium diameter during ventricular systole; LVDD, left ventricular diastolic diameter; LVPWD, diastolic thickness posterior wall of the left ventricle; LVSD, left ventricular systolic diameter; PWSV, posterior wall shortening velocity; RTLV, Relative thickness of the left ventricle; Transmitral flow, Tei‐a and Tei‐b.

**FIGURE 1:**
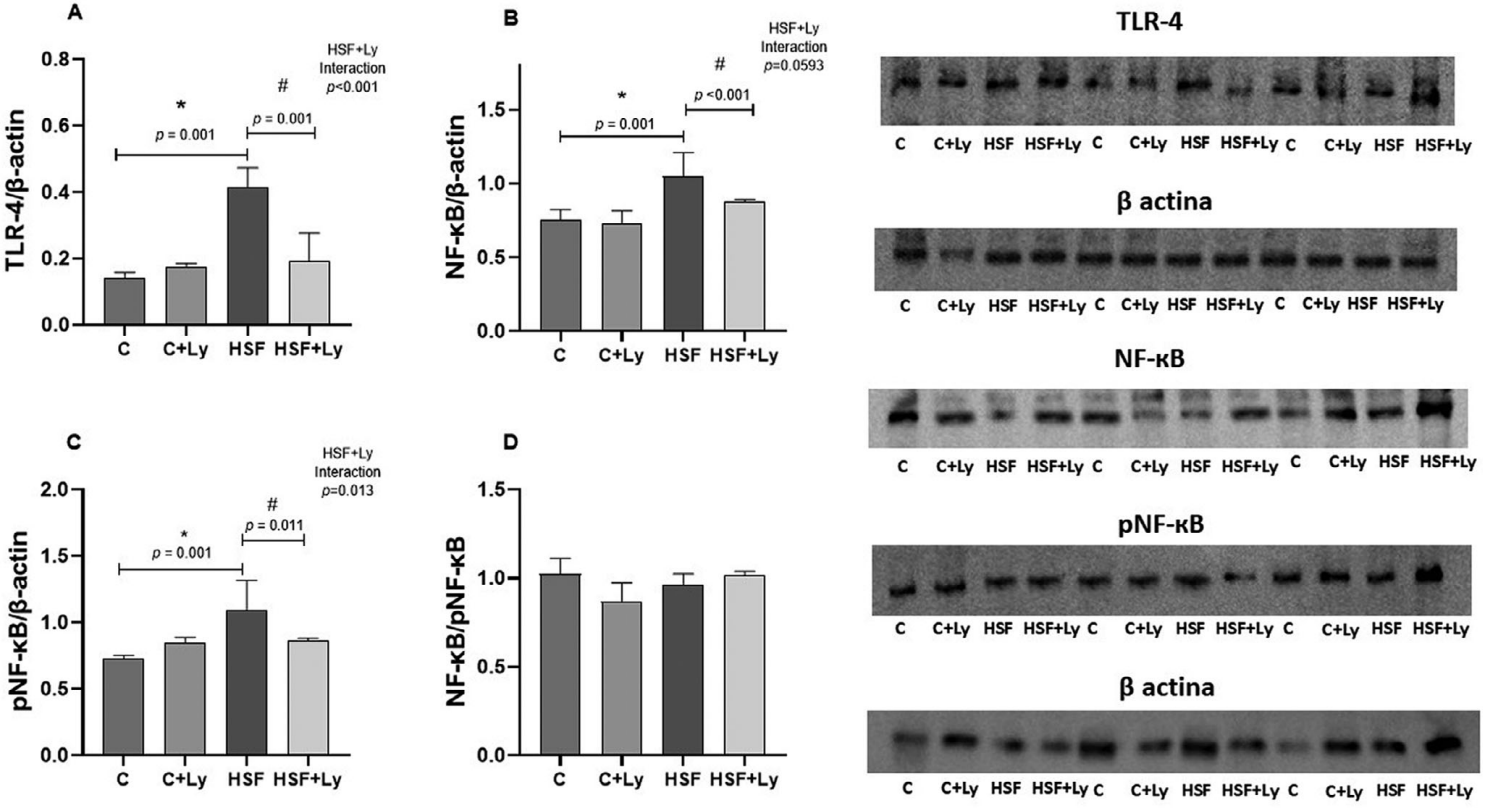
Lycopene supplementation attenuates the expression of inflammatory mediators in the hearts of obese rats. A) Protein expression of TLR‐4 normalized to β‐actin. (B) Protein expression of NF‐κB normalized to β‐actin. (C) Protein expression of phosphorylated NF‐κB (pNF‐κB) normalized to β‐actin. (D) Ratio between total NF‐κB and pNF‐κB. Representative blots for TLR‐4, NF‐κB, pNF‐κB, and β‐actin are shown below the graphs. Data are expressed as mean ± standard deviation (*n* = 8 animals per group). Two‐way ANOVA followed by Tukey's post hoc test was used to compare differences between groups (*p* < 0.05). Results followed by * indicate a significant difference compared with the control group, and results followed by # indicate a significant difference compared with the HSF group. TLR‐4: toll‐like receptor‐4; NF‐κB: nuclear factor kappa B; pNF‐κB: phosphorylated nuclear factor kappa B.

Figure 1 shows the effect of diet and lycopene supplementation on myocardial protein expression of NF‐κB, pNF‐κB, NF‐κB/pNF‐κB ratio, and TLR4. Higher protein expression of NF‐κB, pNF‐κB, and TLR‐4 was observed in the HSF animal group when compared to C (*p* <0.05). Lycopene‐supplemented rats treated with HSF diet displayed a significant decrease in those cytokine protein expressions (HSF+Ly vs. HSF). The NF‐κB/pNF‐κB ratio did not show significant differences among groups.

**FIGURE 2:**
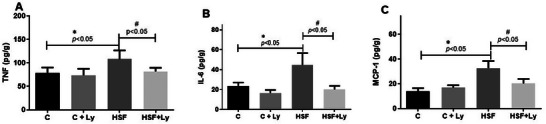
Lycopene supplementation attenuates inflammation in the heart of obese rats through modulation of Inflammatory cardiac mediator levels. TNF‐α, tumor necrosis factor‐α (2.A); IL‐6, interleukin‐6 (2.B); MCP‐1, monocyte chemoattractant protein 1 (2.C). Data expressed as mean ± standard deviation (n, 8 animals / group Two‐way ANOVA with post‐hoc Tukey were used to compare difference between groups (*p* <0.05), results followed by * indicate a significant difference when compared to the control group, and results followed by # indicate a significant difference when compared to the HSF group. TNF‐α, tumor necrosis factor‐α; IL‐6, interleukin‐6; MCP‐1, monocyte chemoattractant protein.

Figure 2 shows the effect of diet and lycopene supplementation on myocardial inflammatory mediator levels. The high sugar fat treatment was associated with a significant increase in TNF‐α, IL‐6, and MCP‐1 (HSF vs. C). Lycopene‐supplemented rats fed the HSF diet displayed a significant decrease in these cytokine levels (HSF + Ly vs. HSF).

**FIGURE 3:**
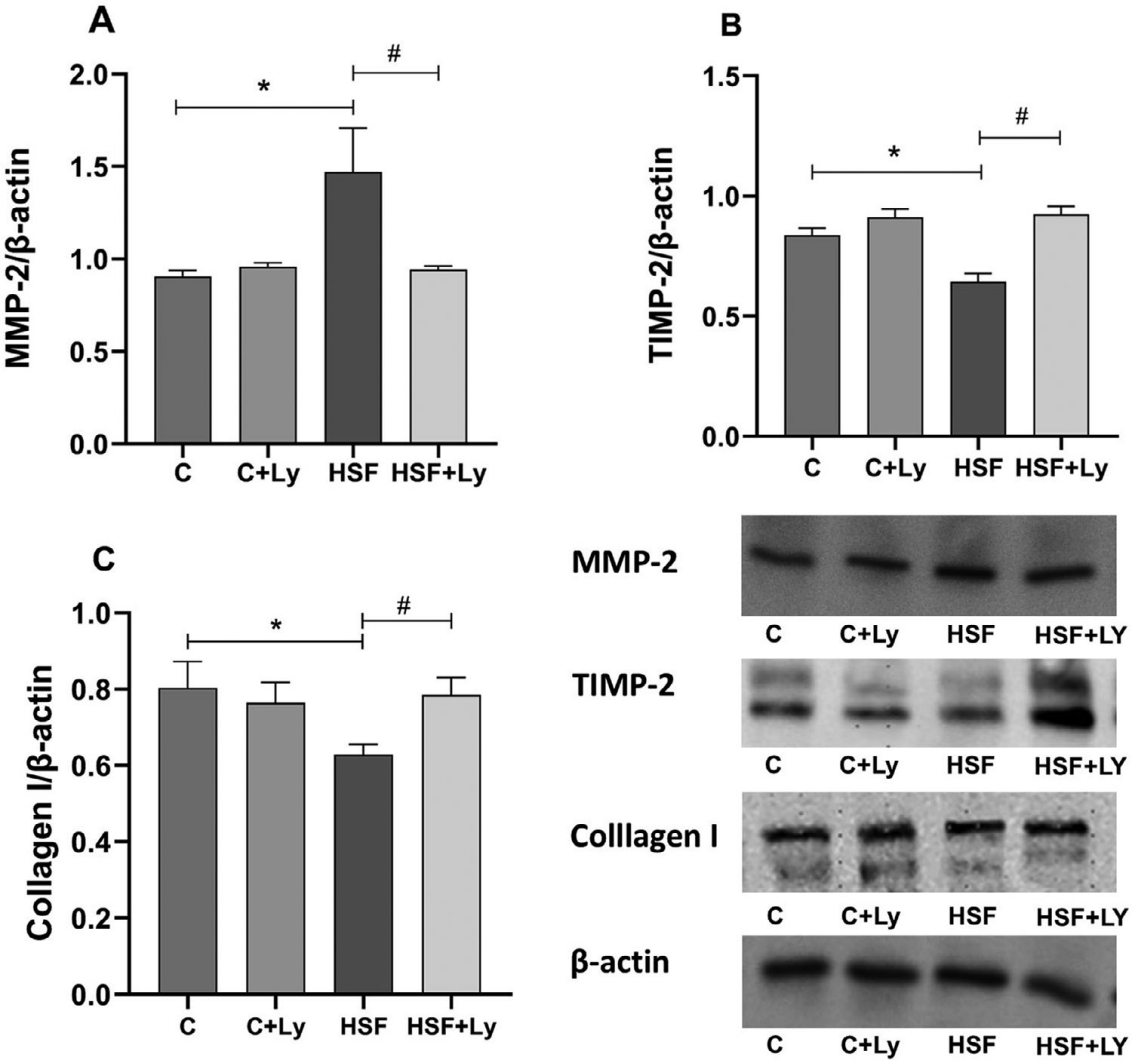
Lycopene supplementation attenuates cardiac MMP‐2 expression, increases TIMP‐2 and Col I in obese rats, contributing to attenuation of cardiac remodeling in obese rats. Data expressed as mean ± standard deviation (n, 8 animals / group). Two‐way ANOVA with post‐hoc Tukey were used to compare difference between groups (*p* <0.05), Results followed by * indicate a significant difference when compared to the control group, and results followed by # indicate a significant difference when compared to the HSF group. MMP‐2, metalloproteinase‐2; TIMP‐2, tissue inhibitor of metalloproteinases–2, Col I, collagen type I.

The high sugar fat treatment affected myocardial protein expression of MMP‐2, TIMP‐2, and Col I. When compared to C animal group, HSF diet was associated with a significant increase in MMP‐2, and decrease in TIMP‐2 and Col I. Conversely, lycopene‐supplemented rats treated with HSF diet improved those parameters (Figure 3).

**FIGURE 4:**
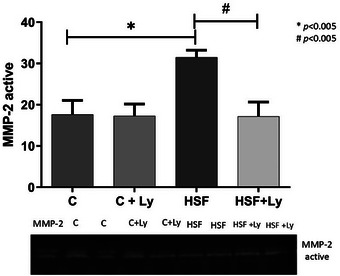
Lycopene supplementation attenuates Active metalloproteinase 2 (active MMP ‐ 2) activity in the heart of obese rats contributing to the attenuation of cardiac remodeling. Data expressed as mean ± standard deviation (n, 8 animals / group). Two‐way ANOVA with post‐hoc Tukey were used to compare difference between groups (*p* <0.05), Results followed by * indicate a significant difference when compared to the control group, and results followed by # indicate a significant difference when compared to the HSF group. Active MMP‐2, metalloproteinase‐2.

It was verified that the activity of metalloproteinase‐2 (active MMP‐2) was higher in HSF animal group, as compared to C. Lycopene‐supplemented rats fed the HSF diet showed reduced active MMP‐2 activity (Figure 4).

**FIGURE 5:**
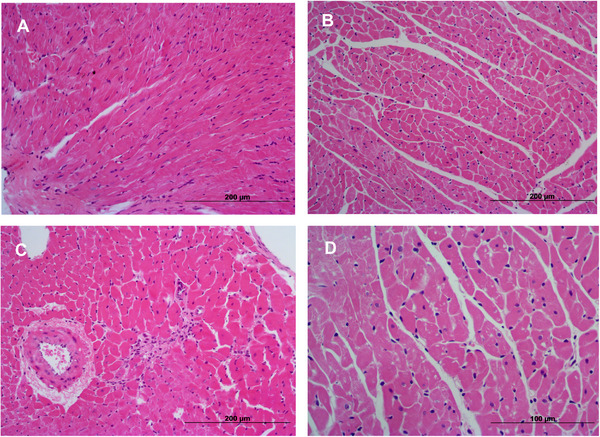
Lycopene supplementation restructures the cardiac tissue in HSF fed rats observed in H&E staining. A, control group (C); B control+lyconepe (C+ly); C,High sugar fat diet (HSF); D,High sugar fat+lycopene (HSF+ly).Control group compared to healthy supplemented one (C + Ly) demonstrated similar morphology. Miocardial fibrosis in cardiac tissue of HSF fed rats compared to reorganized tissue of HSF + Ly. Scale bar: 200 and 100 mm.

The histological images demonstrated that in healthy rats (C), lycopene supplementation (C + Ly) did not induce significant morphological changes compared to the C group (Figure 5A,B). The myocardial fibrosis could be seen in the interstitium of rats LV in the HSF group, indicating tissue remodeling (Figure 5C). When rats with an HSF diet were supplemented with lycopene, a similar morphological profile as described in healthy rats was observed (Figure 5D).

## 4 Discussion

Obesity, a global health challenge, is characterized by excessive body fat accumulation, primarily driven by chronic positive energy balance and hypercaloric dietary intake. Our study confirms that a high sugar‐fat (HSF) diet administered for 30 weeks significantly increased body weight and adiposity index in animals, consistent with established literature on diet‐induced obesity models [4, 6, 24, 25, 31, 38, 39, 40]. This pathological weight gain is intrinsically linked to a cascade of physiological and molecular alterations, predisposing individuals to various cardiometabolic comorbidities. The expansion of adipose tissue, particularly visceral fat, contributes to a state of chronic low‐grade inflammation, a hallmark of obesity‐related pathologies [6, 24, 41]. This inflammatory state, characterized by the release of proinflammatory cytokines, can induce lipotoxicity and inflammation within vital organs, including the myocardium, initiating adverse structural and functional cardiac remodeling [4, 42].

Despite the significant impact of the HSF diet on weight gain, lycopene supplementation for 10 weeks did not significantly alter either body weight or the adiposity index in the treated animals. This observation suggests that, within the context of this study, lycopene primarily exerts its beneficial effects through the modulation of molecular and inflammatory pathways rather than acting as a direct antiobesity agent by reducing fat mass. This aligns with evidence highlighting the pleitropic effects of lycopene,, which extend beyond mere weight reduction to encompass improvements in metabolic and cardiovascular health through its potent antioxidant and anti‐inflammatory properties [21]. The lack of direct impact on weight gain underscores the complexity of obesity pathophysiology and the diverse mechanisms through which bioactive compounds can confer protection against its deleterious consequences.

### 4.1 Inflammation

The inflammatory response plays a central role in the pathogenesis of obesity‐induced cardiac remodeling [7, 43]. Our findings (Figures 1 and 2), demonstrate a significant upregulation of TLR‐4, NF‐κB, and pNF‐κB expression in the HSF group, accompanied by elevated levels of pro‐inflammatory cytokines (TNF‐α, IL‐6, and MCP‐1). These results corroborate the established understanding that diet‐induced obesity robustly activates the TLR‐4/NF‐κB inflammatory pathway, which is central to chronic low‐grade inflammation [6, 41]. Activation of TLR‐4 by free fatty acids and other metabolic by‐products initiates a signaling cascade that activates NF‐κB, the master transcription factor for numerous proinflammatory mediators [10, 42]. This sustained inflammatory state contributes to significantly to systemic and local tissue damage, impacting the cardiovascular system and leading to a pathologic cardiac remodeling [44, 45].

Conversely, lycopene supplementation effectively attenuated these inflammatory alterations. Our data reveal that lycopene significantly diminished TLR‐4 expression, NF‐κB phosphorylation, and the circulating levels of TNF‐α, IL‐6, and MCP‐1 in the HSF+Ly group (Figures 1 and 2). These potent anti‐inflammatory actions primarily attributed to lycopene robust antioxidant capacity and its direct ability to modulate inflammatory signaling pathways [17, 18, 23]. Specifically, lycopene is known to impede NF‐κB activation by suppressing IκBα phosphorylation and inhibiting the nuclear translocation, leading to a substantial reduction in proinflammatory cytokines production [46]. [18, 47, 48] This capacity of lycopene to counteract chronic inflammatory is crucial for preventing and reversing the downstream physiological and structural changes observed in obesity‐related cardiac dysfunction.

### 4.2 Biochemical Profile

Following 30 weeks of the HSF diet, animals exhibited an altered biochemical profile, characterized by elevated glucose, insulin, and HOMA‐IR levels, indicative of developing insulin resistance. Insulin resistance is a pivotal characteristic of obesity and is strongly correlated with an elevated risk of cardiovascular diseases [49]. Both compensatory hyperinsulinemia and chronic hyperglycemia exert direct detrimental effects on the myocardium, fostering endothelial dysfunction and inflammation [4, 49]. These metabolic disturbances activate signaling pathways, such as the mitogen‐activated protein kinase (MAPK) and NF‐κB pathways, which significantly contribute to pathological remodeling [6, 9, 45]. Recent investigations have further elucidated the intricate mechanisms through which insulin resistance and dyslipidemia, commonly associated with obesity [34, 49], contribute to obesity‐induced cardiomyopathy, encompassing alterations in myocardial energy metabolism and lipid accumulation [50].

Lycopene demonstrated a significant effect in reducing insulin and HOMA‐IR levels in the HSF+Ly group after 10 weeks of supplementation, compared with the HSF group, suggesting an improvement in insulin sensitivity. This effect is likely mediated by the modulation of insulin signaling pathways and the attenuation of inflammation, both key contributors to insulin resistance [48, 51]. Lycopene's potent antioxidant properties enable it to scavenge reactive oxygen species, reducing inflammation that impairs insulin signaling [12, 16, 17, 18, 46]. The observed improvement in insulin sensitivity, even without complete normalization of other metabolic parameters, represents a crucial step in mitigating the deleterious effects of obesity. Although lycopene's capacity to modulate glucose metabolism is supported by some studies [18, 48, 51, 52], the lack of a significant effect on glucose levels in this study might be [31, 52, 53] attributable to the specific dose, duration of treatment, or the inherent complexity of HSF diet's interaction with overall metabolism, warranting further investigation [31, 52, 53].

### 4.3 Blood Pressure

The elevation in SBP observed in the HSF group after 30 weeks indicates the development of obesity‐induced hypertension. Obesity contributes to hypertension through a multiple of mechanisms, including activation of the renin–angiotensin–aldosterone system (RAAS), heightened sympathetic activity, endothelial dysfunction, and insulin resistance [54, 55, 56]. The chronic pressure overload from hypertension instigates cardiac adaptations, initially compensatory, but which ultimately progress to pathological remodeling, such as left ventricular hypertrophy [57, 58]. The heart responds to increased afterload by developing left ventricular hypertrophy, a process aimed at normalizing myocardial wall stress. However, prolonged hypertrophy can lead to both diastolic and systolic dysfunction, manifesting as impaired chronotropism and inotropism [59, 60]. The chronic low‐grade inflammation associated with obesity, evidenced by elevated proinflammatory cytokines and activated NF‐κB, exacerbates hypertension by contributing to vascular dysfunction and arterial stiffness [6, 61].

Although lycopene's antioxidant and anti‐inflammatory properties could theoretically influence blood pressure by enhancing endothelial function and reducing arterial stiffness, our findings suggest a parcial protective effect rather than a complete reversal of estabilished hypertension. Lycopene attenuated the increase in SBP in the HSF+Ly group but did not fully normalize it to control levels. Hypertension in obesity is a complex multifactorial phenomenon [54, 56]. It is plausible that lycopene intervention is more effective in preventing hypertension or during the early stages of the disease than reversing a chronic condition. Lycopene's ability to reduce inflammation can improve endothelial nitric oxide bioavailability, which is crucial for vascular relaxation and blood pressure regulation [12, 14, 15, 62].

### 4.4 Cardiac Remodeling

The echocardiographic alterations observed following 30 weeks on the HSF diet collectively signify pathological cardiac remodeling. Our data indicate increased left ventricular systolic diameter (LVSD), left ventricular posterior wall thickness (LVPWT), and reduced left ventricular diastolic diameter (LVDD), coupled with the deterioration of both systolic (decreased ejection fraction (EF) and posterior wall shortening velocity (PWSV) and diastolic (increased E/E’ and Tei index) functions. This remodeling is characterized by concentric hypertrophy, wherein an increase in ventricular wall thickness occurs without a proportional expansion of the cavity, leading to early diastolic dysfunction [7, 40, 55, 58]. Chronic inflammation and the activation of profibrotic signaling pathways contribute to excessive collagen depositon within the myocardial interstitium, culminating in fibrosis and ventricular stiffness [7, 9, 63, 64]. Extracellular matrix dysfunction, mediated by matrix metalloproteinases (MMPs) and their tissue inhibitors (TIMPs), plays an critical role in this pathological process [7, 9, 65, 66]. Ultimately, this pathological remodeling results in compromised cardiac function, frequently culminating in heart failure with preserved ejection fraction (HFpEF), a condition of increasing prevalence among obese patients [1, 50, 59].

Lycopene supplementation was associated with significant improvements in several remodeling parameters, including LVSD, EF, and E/E’, as shown in our results. These findings demonstrate lycopene's capacity to attenuate the progression of pathological cardiac remodeling. The observed improvements in both systolic (EF) and diastolic (E/E’) function suggest that lycopene can effectively preserve myocardial structural and functional integrity. The clear relationship between these echocardiographic improvements and the reduction of inflammation is evident. [53] By modulating these inflammatory pathways, lycopene can mitigate inflammation, fibrosis, and cardiomyocyte apoptosis, which are fundamental mechanisms underlying cardiac remodeling. Lycopene's cardioprotective effects are consistenly demonstrated through the modulation of signaling pathways, enhanced mitochondrial function [17], and reduced inflammation and fibrosis [21, 46].

### 4.5 Histological Changes

The histological data provide compelling visual evidence of lycopene's cardioprotective effects against diet‐induced pathological remodeling. As seen in Figure 5C, HSF diet induced significant myocardial fibrosis within the left ventricular interstitium, a hallmark of pathological tissue remodeling that supports increased ventricular stiffness and dysfunction [67]. Conversely, lycopene supplementation in HSF‐fed rats markedly attenuated this fibrotic response, restoring a morphological profile (Figure 5D) comparable to that of healthy, normotrophic hearts (Figures 5A,B). This structural preservation is mechanistically coupled with the observed modulation of ECM homeostasis [9]. The HSF diet disrupted the delicate balance of ECM turnover by heightening the activity of MMP‐2, while concurrently suppressing its endogenous inhibitor, TIMP‐2, and reducing type I collagen expression. Lycopene's ability to normalize the myocardial architecture is therefore directly attributable to its capacity to counteract these molecular changes, specifically by inhibiting MMP‐2 activity and restoring TIMP‐2 and collagen levels. Notably, the absence of morphological alterations in healthy rats receiving lycopene (C + Ly group) underscores the specificity of its therapeutic action, which selectively targets pathological pathways without perturbing normal cardiac tissue physiology.

### 4.6 MMP‐2 Pathway

The ECM plays a crucial role in maintaining cardiac structure and function, and its dysregulation is a key feature of pathological cardiac remodeling [1, 7, 43]. Our results (Figures 3 and 4) demonstrate that the HSF group exhibited a significant increase in MMP‐2 expression and activity, a concomitant reduction in TIMP‐2 expression, and a decrease in type I collagen expression. These alterations are characteristic of an imbalance in ECM homeostasis, leading to excessive collagen degradation and myocardial fibrosis [9, 65, 68]. MMP‐2, a gelatinase, plays a central role in pathological cardiac remodeling by degrading various ECM components, contributing to ventricular dysfunction and stiffness [9, 66]. [69, 70] The imbalance between MMPs and TIMPs, with decrease of TIMP‐2, shifts the balance toward degradation promoting pathological cardiac remodeling and contributing to the echocardiographic alterations and impaired cardiac function observed.

Lycopene supplementation significantly reversed these detrimental changes in ECM components. Our data show that lycopene reduced MMP‐2 expression and activity, increased TIMP‐2 expression, and restored type I collagen expression in the HSF+Ly group (Figures 3 and 4). These findings demonstrate that lycopene is capable of modulating MMP activity and collagen synthesis, thereby fostering a healthier balance within the ECM. Lycopene's ability to inhibit MMP‐2 and restore TIMP‐2 and type I collagen expression represents a key mechanism by which it attenuates pathological cardiac remodeling and fibrosis. This cardioprotective effect is largely attributed to its potent antioxidant and anti‐inflammatory properties, which directly counteract the triggers for MMP activation and ECM degradation. Lycopene acts primarily by mitigating oxidative stress and inflammation caused by a high sugar free diet, rather than altering the basal morphology of already healthy tissue [31, 53]. Then, the observation of any morphological alterations between the control groups was expected. A decrease in fibrosis when rats fed HSF with Ly modulated the expression of profibrotic mediators, resulting in the alteration of Col I deposition in the interstitium. [71, 72, 73, 74], highlighting its therapeutic potential in mitigating obesity‐induced cardiac dysfunction.

### 4.7 Clinical Implications

Despite these significant findings, it is crucial to acknowledge the limitations of the current study. Specifically, the exclusive use of male animals and the relatively short 10‐week intervention period limit the generalizability of our findings, particularly regarding sex‐specific differences and long‐term efficacy. Therefore, caution must be exercised when extrapolating these results from a rat model to human clinical scenarios. Future research should focus on long‐term follow‐up studies, the inclusion of female cohorts to assess sex‐dependent effects, and ultimately, randomized controlled trials to achieve clinical validation of lycopene's therapeutic potential in mitigating obesity‐induced cardiomyopathy.

### 4.8 Conclusion

Our study demonstrates that lycopene supplementation mitigates obesity‐induced cardiac remodeling by modulating inflammatory responses and inhibiting MMP‐2 activation, ultimately leading to enhanced cardiac function and preserved myocardial structure. The novel contribution of this research lies in its elucidation of lycopene's direct impact on MMP‐2, thereby providing a more refined understanding of its cardioprotective mechanisms. These compelling findings lay the groundwork for subsequent investigations into lycopene as a promising therapeutic agent for obesity‐related cardiac disorders, particularly those characterized by dysregulation of the ECM

**Use of AI tools: ChatGPT (OpenAI) was employed solely for grammar verification and minor language refinement. The scientific content, data interpretation, and conclusions were entirely developed by the authors**.

## Funding

This work was funded by Conselho Nacional de Desenvolvimento Científico e Tecnológico CNPq (424209/2016‐0) and Cordenaçao de Aperfeiçoamento de Pessoal de Nivel superior ‐ CAPES.

## Conflicts of Interest

The authors declare no conflicts of interest.

## Data Availability

The data that support the findings of this study are available from the corresponding author upon reasonable request.
